# Kahweol Ameliorates Cisplatin-Induced Acute Kidney Injury through Pleiotropic Effects in Mice

**DOI:** 10.3390/biomedicines8120572

**Published:** 2020-12-06

**Authors:** Jung-Yeon Kim, Jungmin Jo, Jaechan Leem, Kwan-Kyu Park

**Affiliations:** 1 Department of Immunology, School of Medicine, Catholic University of Daegu, Daegu 42472, Korea; jy1118@cu.ac.kr; 2 Division of Hematology-Oncology, Department of Internal Medicine, Ewha Womans University Mokdong Hospital, Seoul 07985, Korea; 10003kj@ewha.ac.kr; 3 Department of Pathology, School of Medicine, Catholic University of Daegu, Daegu 42472, Korea; kkpark@cu.ac.kr

**Keywords:** cisplatin, coffee, kahweol, acute kidney injury, oxidative stress, apoptosis, necroptosis, inflammation

## Abstract

Cisplatin is an effective chemotherapeutic agent, but its clinical use is frequently limited by its nephrotoxicity. The pathogenesis of cisplatin-induced acute kidney injury (AKI) remains incompletely understood, but oxidative stress, tubular cell death, and inflammation are considered important contributors to cisplatin-induced renal injury. Kahweol is a natural diterpene extracted from coffee beans and has been shown to possess anti-oxidative and anti-inflammatory properties. However, its role in cisplatin-induced nephrotoxicity remains undetermined. Therefore, we investigated whether kahweol exerts a protective effect against cisplatin-induced renal injury. Additionally, its mechanisms were also examined. Administration of kahweol attenuated renal dysfunction and histopathological damage together with inhibition of oxidative stress in cisplatin-injected mice. Increased expression of nicotinamide adenine dinucleotide phosphate oxidase 4 and decreased expression of manganese superoxide dismutase and catalase after cisplatin treatment were significantly reversed by kahweol. Moreover, kahweol inhibited cisplatin-induced apoptosis and necroptosis in the kidneys. Finally, kahweol reduced inflammatory cytokine production and immune cell accumulation together with suppression of nuclear factor kappa-B pathway and downregulation of vascular adhesion molecules. Together, these results suggest that kahweol ameliorates cisplatin-induced renal injury via its pleiotropic effects and might be a potential preventive option against cisplatin-induced nephrotoxicity.

## 1. Introduction

Cisplatin is a platinum-containing chemotherapeutic agent that has been widely used for the treatment of various human cancers [1]. However, its clinical use is frequently limited due to its side effects. Among them, acute kidney injury (AKI) is the most common dose-limiting side effect of cisplatin treatment. Indeed, about a third of patients on cisplatin treatment suffer from the nephrotoxic side effect [1]. However, no effective therapies for cisplatin-induced AKI are currently available. Thus, it is essential to develop therapeutic agents for preventing nephrotoxicity, enabling high-dose chemotherapy using cisplatin.

The pathophysiology of cisplatin-induced renal injury involves multiple mechanisms that are incompletely understood. However, oxidative stress, tubular cell death, and inflammation are considered important contributors to cisplatin-induced renal injury [1,2,3]. Among them, high levels of oxidative stress are one of the hallmarks of cisplatin-induced renal injury. Numerous studies have reported that cisplatin treatment is associated with increased generation of reactive oxygen species (ROS) and decreased expression of endogenous antioxidant enzymes [4,5]. High levels of oxidative stress can induce tubular cell death [6]. Previous studies have shown that apoptosis of tubular epithelial cells occurs in cisplatin-induced renal injury [1,2,3]. Apoptosis is a form of programmed cell death and is mediated by caspases, which trigger cell death by catalyzing the specific cleavage of numerous essential cellular proteins. In addition, necrosis, a non-programmed cell death, is also observed in kidneys of rodents treated with cisplatin [1,2,3]. Recently, a novel type of programmed necrosis, called necroptosis, has emerged as another important type of cell death in cisplatin-induced AKI [7,8]. Indeed, concomitant use of inhibitors of apoptosis and necroptosis presented synergistic renoprotective effects against cisplatin-induced renal injury [9]. Besides direct cellular damage, inflammatory responses are also critical for cisplatin-induced renal injury [1,2,3]. During cisplatin-induced AKI, excessive amounts of cytokines are produced and secreted from infiltrated pro-inflammatory cells and tubular epithelial cells. Genetic or pharmacological suppression of tumor necrosis factor-α (TNF-α) effectively protected against cisplatin-induced renal injury in mice [10]. Because tubular epithelial cells also secrete a variety of chemokines during cisplatin-induced AKI, pro-inflammatory cells, including neutrophils, macrophages, and CD4^+^ T cells, are infiltrated into damaged kidneys [11,12]. Excessive accumulation of pro-inflammatory cells can induce additional tissue injury via the generation of cytokines and ROS.

Coffee is the most popularly consumed beverage all over the world after water. A number of epidemiological studies have shown that coffee consumption is inversely proportional to the risk of various human diseases such as cardiovascular disease, cancer, diabetes, and chronic liver disease [13]. Coffee contains a variety of biologically active components. Among them, kahweol is a natural diterpene extracted from coffee beans and has been shown to exhibit anti-oxidative and anti-inflammatory activities [14]. Accumulating evidence suggests that the compound has a beneficial effect on inflammatory conditions of various organs [15,16,17]. However, whether kahweol has a beneficial effect against cisplatin-induced renal injury has not yet been clarified. The purpose of this study was to evaluate the potential effects of kahweol on cisplatin-induced renal injury and to explore its mechanisms.

## 2. Materials and Methods

### 2.1. Animals Procedures

All animal experiments were performed in accordance with the Institutional Animal Care and Use Committee of the Daegu Catholic University Medical Center (Approval number: DCIAFCR-200626-12-Y, approval date: 6 June 2020). Male 7-week-old C57BL/6N mice were acquired from HyoSung Science Inc. (Daegu, Korea) and kept at 20–24 °C and 55% humidity for 1 week. The mice were divided into 3 groups (*n* = 8 per group): vehicle (Veh), cisplatin (CP), and cisplatin plus kahweol (CP+Kah). The CP group was given a single intraperitoneal injection of cisplatin (20 mg/kg in 0.9% saline). An equal volume of the vehicle was injected intraperitoneally into the Veh group. The CP+Kah group was given an intraperitoneal injection of kahweol (20 mg/kg) daily for 4 consecutive days, starting from 1 day prior to cisplatin injection. The doses of kahweol and cisplatin and the treatment protocol were selected based on the results of previous studies [15,18]. Cisplatin was purchased from Sigma-Aldrich (St. Louis, MO, USA) and kahweol was obtained from Abcam (Cambridge, MA, USA). All mice were sacrificed 72 h after cisplatin injection.

### 2.2. Biochemical Analysis

Plasma creatinine and blood urea nitrogen (BUN) levels were analyzed using a creatinine assay kit (BioAssay Systems, Hayward, CA, USA) and a BUN assay kit (Thermo Fisher Scientific, Waltham, MA, USA), respectively, according to the manufacturer’s protocol. A creatinine value more than 0.5 mg/dL [19] or a BUN value more than 33 mg/dL [20] was considered as acute renal failure. Plasma TNF-α and interleukin-6 (IL-6) levels were measured using standard quantitative sandwich ELISA kits (R&D Systems, Minneapolis, MN, USA) according to the manufacturer’s protocol. Renal malondialdehyde (MDA) levels were measured using a colorimetric/fluorometric assay kit (Sigma-Aldrich, St. Louis, MO, USA) according to the manufacturer’s protocol. Renal levels of reduced glutathione (GSH) and oxidized glutathione (GSSG) were analyzed using a colorimetric detection kit (Enzo Life Sciences, Farmingdale, NY, USA) according to the manufacturer′s protocol.

### 2.3. Histological Analysis, Immunohistochemical Staining, and Immunofluorescent Staining

Isolated kidney tissues were immediately fixed in 10% formalin and then dehydrated in graded series of ethanol. After dehydration, the tissues were cleared in xylene and embedded in paraffin. Thin sections (4 μm) were mounted on glass slides and stained with hematoxylin and eosin (H&E) or periodic acid-Schiff (PAS). The severity of tubular injury was scored semiquantitatively by estimating the percentage of damaged area: 0, 0%; 1, ≤10%; 2, 11–25%; 3, 26–45%; 4, 46–75%; and 5, 76–100% [21,22]. Tubular injury was assessed in 5 arbitrarily chosen fields at ×400 magnification per kidney sample. For immunohistochemical staining, the sections were incubated with a primary antibody overnight and then probed with a secondary antibody. The primary antibodies used for immunohistochemical staining were as follows: anti-neutrophil gelatinase-associated lipocalin (NGAL; Santa Cruz Biotechnology, Santa Cruz, CA, USA), anti-kidney injury molecule-1 (KIM-1; Abcam, Cambridge, MA, USA), anti-galectin-3 (Abcam, Cambridge, MA, USA), anti-CD4 (Abcam, Cambridge, MA, USA), or anti-4-hydroxynonenal (4-HNE; Abcam, Cambridge, MA, USA) antibodies. Images were visualized and captured using a confocal microscope (Nikon, Tokyo, Japan). The percentage of stained areas was determined in 5 arbitrarily chosen fields at ×400 magnification per kidney sample using the i-Solution DT software (IMTechnology, Vancouver, BC, Canada). The number of cells stained with anti-galectin-3 or anti-CD4 antibody was counted in 5 arbitrarily chosen fields at ×400 magnification per kidney sample.

Lotus tetragonolobus lectin (LTL) is a well-known marker for detecting the proximal tubule brush border [22]. The kidneys sections were stained with fluorescein isothiocyanate (FITC)-labeled LTL (Vector Laboratories, Burlingame, CA, USA). Additionally, to identify neutrophils, the sections were probed with anti-Ly6B.2 antibody (Abcm, Cambridge, MA, USA) and then incubated with a secondary antibody. The percentage of stained areas or the number of cells stained with anti-Ly6B.2 antibody was determined in 5 arbitrarily selected fields at ×400 magnification per kidney sample. To stain nuclei, 4′,6-diamidino-2-phenylindole (DAPI) was used.

### 2.4. Western Blot Analysis

Total proteins were extracted from kidney tissues with a lysis buffer and then loaded onto precast gradient polyacrylamide gels (Thermo Fisher Scientific, Waltham, MA, USA). Separated proteins were transferred from gels to nitrocellulose membranes. The membranes were probed with primary antibodies against nicotinamide adenine dinucleotide phosphate oxidase 4 (NOX4), catalase, manganese superoxide dismutase (MnSOD), cleaved caspase-3, cleaved poly(ADP-ribose) polymerase-1 (cleaved PARP-1), receptor-interacting serine/threonine protein kinase 1 (RIPK1), RIPK3, mixed lineage kinase domain-like protein (MLKL), p-MLKL, nuclear factor-κB (NF-κB) p65, p-NF-κB p65, intercellular adhesion molecule-1 (ICAM-1), and glyceraldehyde-3-phosphate dehydrogenase (GAPDH), followed by incubation with horseradish peroxidase-conjugated secondary antibodies. Primary antibodies against cleaved caspase-3, cleaved PARP-1, RIPK1, RIPK3, MLKL, p65, p-p65, and GAPDH were purchased from Cell Signaling (Danvers, MA, USA). Primary antibodies against catalase, MnSOD, and p-MLKL were acquired from Abcam. Anti-NOX4 antibody was obtained from Novus Biologicals (Littleton, CO, USA), and anti-ICAM-1 antibody was purchased from Santa Cruz Biotechnology. GAPDH was used as an internal control. The protein bands were visualized using enhanced chemiluminescence (ECL) reagents (Thermo Fisher Scientific, Waltham, MA, USA). The signal intensity was analyzed using the iBright™ CL1500 Imaging System (Thermo Fisher Scientific, Waltham, MA, USA).

### 2.5. Real-Time Reverse Transcription-Polymerase Chain Reaction (RT-PCR)

Total RNA was extracted from kidney tissues using the TRIzol reagent (Thermo Fisher Scientific, Waltham, MA, USA). Reverse transcription was carried out using the RNA to cDNA EcoDry™ Premix Kit (TaKaRa, Tokyo, Japan) according to the manufacturer′s protocol. Real-time RT-PCR reactions were performed using the Thermal Cycler Dice Real Time System III (TaKaRa, Tokyo, Japan) and the Power SYBR Green PCR Master Mix (TaKaRa, Tokyo, Japan). Primers used in this study are listed in Table 1. The internal reference gene was GAPDH.

### 2.6. TdT-Mediated dUTP Nick End Labeling (TUNEL) Assay

Apoptosis was assessed using a TUNEL assay kit (Roche Diagnostics, Indianapolis, IN, USA) according to the manufacturer’s protocol. Nuclei were stained with DAPI. Images were visualized and captured using a confocal microscope (Nikon, Tokyo, Japan). The number of cells stained with TUNEL was counted in 5 randomly chosen fields at ×400 magnification per kidney sample.

### 2.7. Statistical Analysis

Data are presented as mean ± standard error of the mean (SEM) and analyzed with one-way analysis of variance (ANOVA), which was followed by Bonferroni’s *post hoc* tests. A *p* value less than 0.05 was considered statistically significant.

## 3. Results

### 3.1. Kahweol Attenuated Cisplatin-Induced Kidney Injury

**Figure 1:**
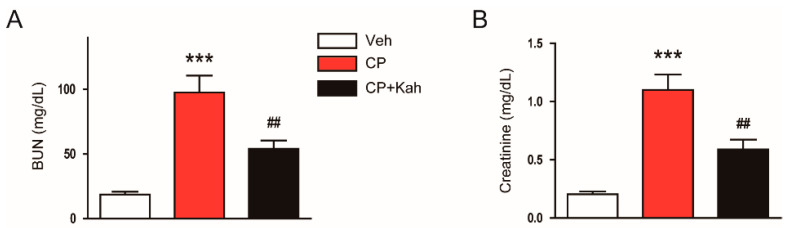
Effect of kahweol on plasma blood urea nitrogen (BUN) and creatinine levels in cisplatin-injected mice. Mice were given an intraperitoneal administration with kahweol (20 mg/kg; Kah) daily for 4 consecutive days, starting from 1 day prior to cisplatin injection. All mice were sacrificed 72 h after cisplatin injection and the blood was collected. (**A**) BUN levels. (**B**) Plasma creatinine levels. *n* = 8 per group. *** *p* < 0.001 vs. the vehicle-treated control group (Veh). ^##^ *p* < 0.01 vs. the cisplatin-injected group (CP).

Mice were administered 20 mg/kg of cisplatin for inducing AKI. All mice survived until 72 h after cisplatin injection. As shown in Figure 1A,B, cisplatin treatment increased plasma BUN and creatinine levels, indicating the development of acute renal failure in cisplatin-injected mice. However, administration of kahweol largely decreased the elevated levels of both markers of renal function (Figure 1A,B).

**Figure 2:**
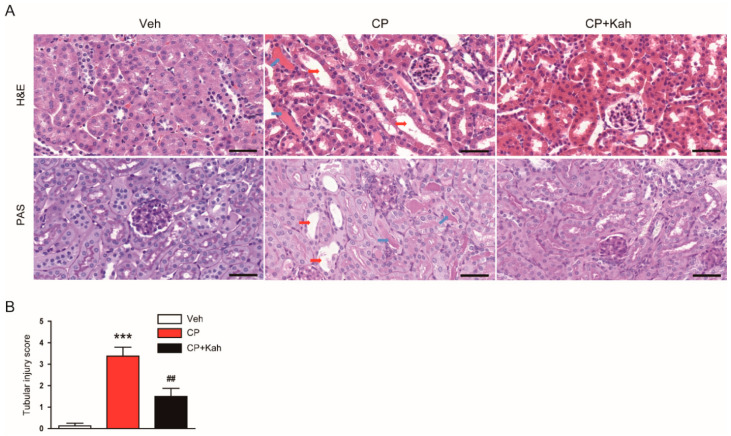
Histological features of the kidneys in all experimental groups. (**A**) Hematoxylin and eosin (H&E) and periodic acid-Schiff (PAS) staining of kidney tissues. Scale bar = 40 μm. Red arrows indicate dilated tubules. Blue arrows indicate tubular cast deposition. (**B**) Tubular injury score. n = 8 per group. *** *p* < 0.001 vs. Veh. ^##^ *p* < 0.01 vs. CP.

Next, the effects of kahweol on cisplatin-induced histological changes were analyzed. H&E and PAS staining showed that cisplatin-injected mice exhibited obvious histological injury, as reflected by tubular dilation and cast formation (Figure 2A,B). These histological alterations were significantly attenuated by kahweol (Figure 2A,B).

**Figure 3:**
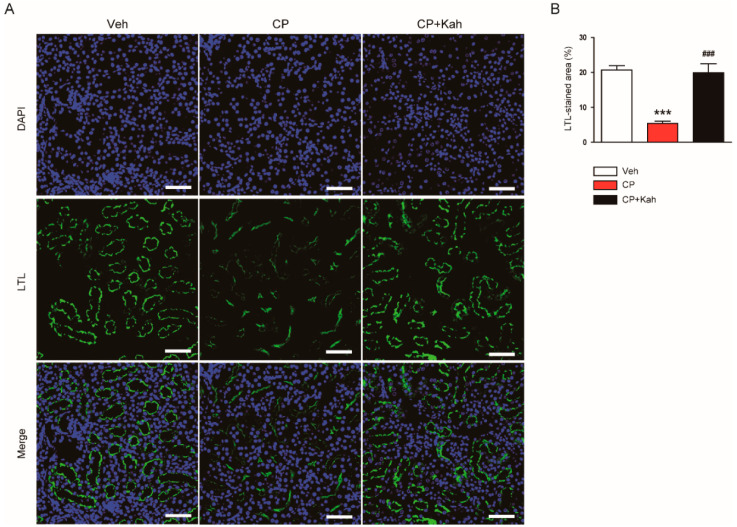
Effect of kahweol on brush border in proximal tubules. (**A**) Lotus tetragonolobus lectin (LTL) staining of kidney tissues. Nuclei were stained with 4′,6-diamidino-2-phenylindole (DAPI). Scale bar = 50 μm. (**B**) Percentage of stained areas for LTL. *n* = 8 per group. *** *p* < 0.001 vs. Veh. ^###^ *p* < 0.001 vs. CP.

Brush border loss in proximal tubules after cisplatin treatment was also demonstrated by LTL staining (Figure 3A,B). However, the administration of kahweol significantly alleviated the brush border loss (Figure 3A,B).

**Figure 4:**
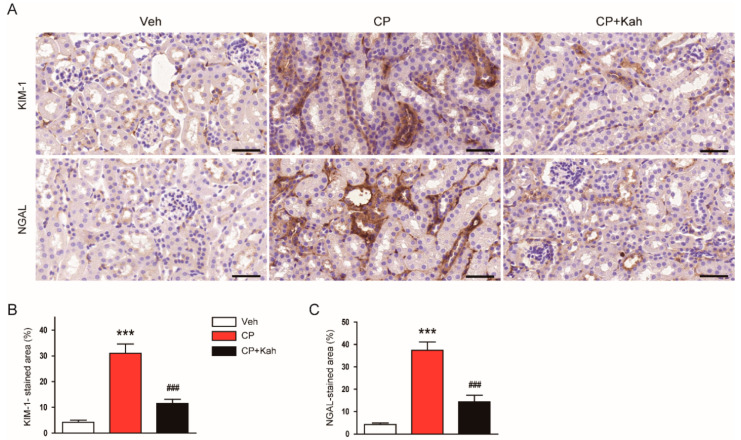
Effect of kahweol on tubular injury markers in cisplatin-injected mice. (**A**) Immunohistochemical staining of kidney tissues for kidney injury molecule-1 (KIM-1) or neutrophil gelatinase-associated lipocalin (NGAL). Scale bar = 50 μm. (**B**) Percentage of stained area for KIM-1. (**C**) Percentage of stained areas for NGAL. *n* = 8 per group. *** *p* < 0.001 vs. Veh. ^###^ *p* < 0.001 vs. CP.

Further, expression of KIM-1 and NGAL, tubular injury markers, was examined using immunohistochemical staining. Cisplatin treatment markedly increased renal expression of both markers (Figure 4A–C). These changes with cisplatin treatment were reduced after administration of kahweol (Figure 4A–C). Together, these results suggest that administration of kahweol ameliorated functional and structural renal injury, especially tubular injury, after cisplatin treatment.

### 3.2. Kahweol Inhibited Cisplatin-Induced Oxidative Stress

**Figure 5:**
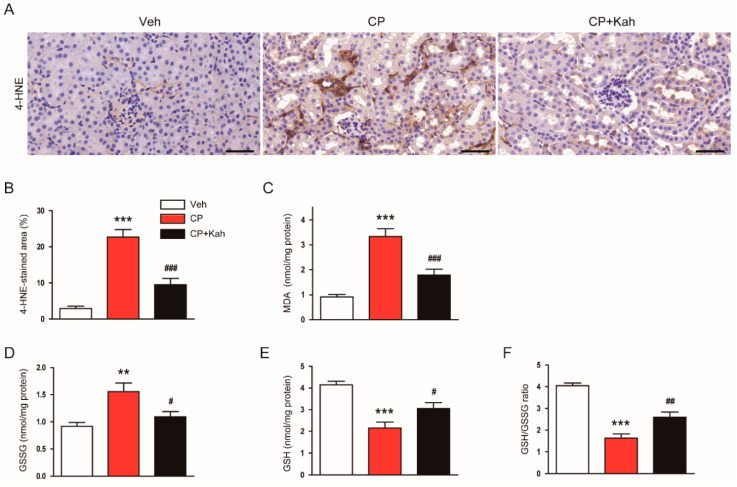
Effect of kahweol on cisplatin-induced renal oxidative stress. (**A**) Immunohistochemical staining of kidney tissues for 4-hydroxynonenal (4-HNE). Scale bar = 100 μm. (**B**) Percentage of stained areas for 4-HNE. (**C**) Malondialdehyde (MDA) levels. (**D**) Oxidized glutathione (GSSG) levels. (**E**) Reduced glutathione (GSH) levels. (**F**) GSH/GSSG ratio. *n* = 8 per group. ** *p* < 0.01 and *** *p* < 0.001 vs. Veh. ^#^ *p* < 0.05, ^##^ *p* < 0.01, and ^###^ *p* < 0.001 vs. CP.

Oxidative injury is a hallmark of cisplatin-induced renal injury [1,2,3]. Previous studies have shown that kahweol has anti-oxidant effects [23,24]. To investigate the effects of kahweol on oxidative stress in kidney tissues of cisplatin-injected mice, the kidney sections were stained with anti-4-HNE antibody. 4-HNE is a by-product of lipid peroxidation and is widely used as a marker of oxidative stress [25,26]. As shown in Figure 5A,B, the percentage of the 4-HNE-stained area was markedly increased after cisplatin treatment. Renal levels of MDA (Figure 5C) and GSSG (Figure 5D) were also increased in the cisplatin-injected mice. However, these increases were significantly alleviated by the administration of kahweol (Figure 5A–D). In addition, kahweol attenuated GSH depletion (Figure 5E) and a reduction of GSH/GSSG ratio (Figure 5F) in the kidneys of cisplatin-injected mice.

**Figure 6:**
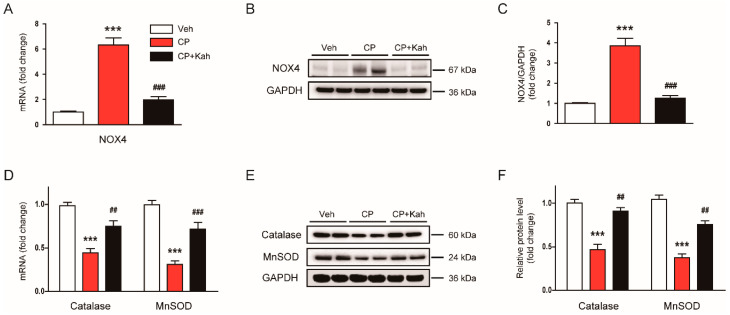
Effect of kahweol on oxidative stress-related enzymes in cisplatin-injected mice. (**A**) The mRNA levels of nicotinamide adenine dinucleotide phosphate oxidase 4 (NOX4). (**B**) Western blotting of NOX4. (**C**) Quantification of Western blot for NOX4. Glyceraldehyde-3-phosphate dehydrogenase (GAPDH) was used as an internal control. (**D**) The mRNA levels of catalase and manganese superoxide dismutase (MnSOD). (**E**) Western blotting of catalase and MnSOD. (**F**) Quantification of Western blots for catalase and MnSOD. GAPDH was used as an internal control. *n* = 8 per group. *** *p* < 0.001 vs. Veh. ^##^ *p* < 0.001 and ^###^ *p* < 0.001 vs. CP.

It has been shown that NOX4 is a major source of ROS in the kidney and plays a critical role in kidney diseases including cisplatin-induced AKI [27,28]. Thus, we next evaluated the effects of kahweol on NOX4 expression. Elevated mRNA (Figure 6A) and protein (Figure 6B,C) levels of NOX4 after cisplatin injection were largely reduced by kahweol. Further, decreased mRNA (Figure 6D) and protein (Figure 6E,F) expression of antioxidant enzymes, catalase, and MnSOD after cisplatin injection was also largely restored by kahweol.

### 3.3. Kahweol Suppressed Cisplatin-Induced Tubular Cell Death

**Figure 7:**
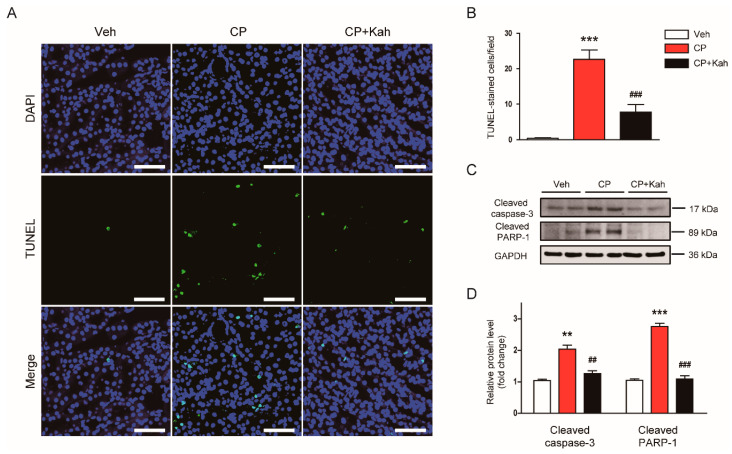
Effect of kahweol on apoptotic cell death in cisplatin-injected mice. (**A**) TdT-mediated dUTP nick end labeling (TUNEL) assay on kidney tissues. Scale bar = 50 μm. To stain nuclei, DAPI was used. (**B**) Number of positively stained cells. (**C**) Western blotting of the proteolytic cleavage of caspase-3 and poly(ADP-ribose) polymerase-1 (PARP-1) proteins. (**D**) Quantification of Western blots for the proteolytic cleavage of caspase-3 and PARP-1. GAPDH was used as an internal control. *n* = 8 per group. ** *p* < 0.01 and *** *p* < 0.001 vs. Veh. ^##^ *p* < 0.01 and ^###^ *p* < 0.001 vs. CP.

Tubular cell apoptosis also plays a crucial role in cisplatin-induced renal injury [1,2,3]. Therefore, we next examined the effects of kahweol on apoptotic death of tubular epithelial cells in cisplatin-injected mice. A marked increase in the number of cells stained with TUNEL was observed after cisplatin treatment (Figure 7A,B). However, kahweol inhibited the cisplatin-induced tubular cell apoptosis, as demonstrated by a reduction in the number of cells stained with TUNEL (Figure 7A,B). Increased cleavage of caspase-3 and PARP-1 were also largely decreased by kahweol, indicating that kahweol suppressed caspase-3 activation (Figure 7C,D).

**Figure 8:**
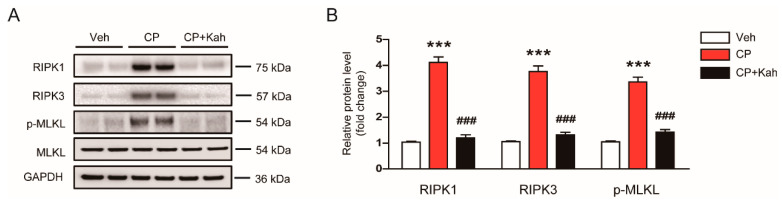
Effect of kahweol on tubular cell necroptosis in cisplatin-injected mice. (**A**) Western blotting of receptor-interacting serine/threonine protein kinase 1 (RIPK1), RIPK3, mixed-lineage kinase domain-like protein (MLKL), and p-MLKL. (**B**) Quantification of Western blots for RIPK1, RIPK3, and p-MLKL. GAPDH was used as an internal control. *n* = 8 per group. *** *p* < 0.001 vs. Veh. ^###^ *p* < 0.001 vs. CP.

Emerging evidence has suggested that necroptosis, a programmed necrosis, also plays a critical role in the pathophysiology of cisplatin-induced AKI [7,8]. To evaluate the effects of kahweol on cisplatin-induced necroptosis, protein levels of RIPK1, RIPK3, and p-MLKL were analyzed by Western blot analysis. Cisplatin treatment largely increased protein expression of RIPK1, RIPK3, and p-MLKL in kidneys (Figure 8A,B). However, this change was significantly inhibited by kahweol (Figure 8A,B).

### 3.4. Kahweol Alleviated Cisplatin-Induced Inflammatory Responses

**Figure 9:**
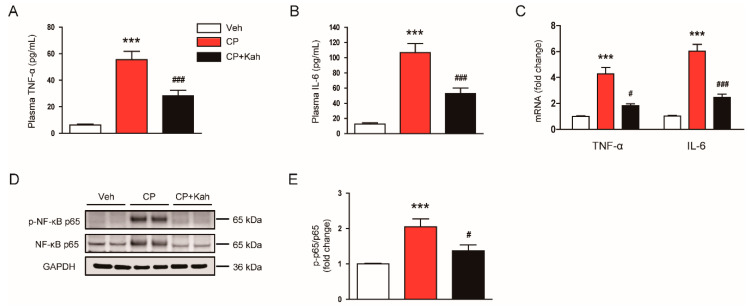
Effect of kahweol on inflammatory cytokine production and nuclear factor-κB (NF-κB) signaling in cisplatin-injected mice. (**A**) Plasma tumor necrosis factor-α (TNF-α) levels. (**B**) Plasma interleukin-6 (IL-6) levels. (**C**) The mRNA levels of TNF-α and IL-6. (**D**) Western blotting of p-NF-κB p65. (**E**) Quantification of Western blot for p-NF-κB p65. GAPDH was used as an internal control. *n* = 8 per group. *** *p* < 0.001 vs. Veh. ^#^ *p* < 0.05 and ^###^ *p* < 0.001 vs. CP.

Accumulating evidence suggests that cisplatin treatment triggers an acute inflammatory response by inducing the secretion of cytokines in immune cells [1,2,3]. Kahweol has also been known to display anti-inflammatory effects [15,16,17]. Thus, we next examined the effects of kahweol on an inflammatory response induced by cisplatin. Cisplatin treatment increased plasma levels of TNF-α and IL-6 (Figure 9A,B), indicating cisplatin-induced systemic inflammation. Renal mRNA levels of these cytokines were also markedly increased in cisplatin-injected mice (Figure 9C). However, these increases were significantly attenuated by kahweol (Figure 9A–C). Because NF-κB plays an essential role in the production of cytokines, we next examined the effects of kahweol on NF-κB signaling. Khaweol significantly reduced the levels of phosphorylated NF-κB p65 in the kidneys of cisplatin-injected mice (Figure 9D,E). Collectively, these results suggest that kahweol suppressed cisplatin-induced systemic and local inflammation.

**Figure 10:**
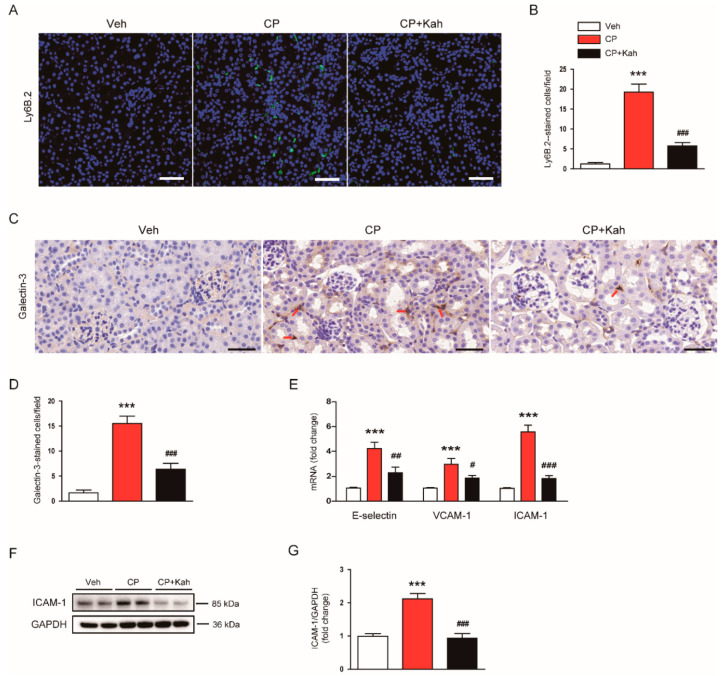
Effect of kahweol on immune cell accumulation in cisplatin-injected mice. (**A**) Immunofluorescent staining of kidney tissues for Ly6B.2. Scale bar = 50 μm. (**B**) Number of Ly6B.2-stained cells. (**C**) Immunohistochemical staining of kidney tissues for galectin-3. Red arrows indicate positively stained cells. Scale bar = 100 μm. (**D**) Number of galectin-3-stained cells. (**E**) The mRNA levels of E-selectin, vascular cell adhesion molecule-1 (VCAM-1), and intercellular adhesion molecule-1 (ICAM-1). (**F**) Western blotting of ICAM-1. (**G**) Quantification of Western blot for ICAM-1. GAPDH was used as an internal control. *n* = 8 per group. *** *p* < 0.001 compared with Veh. ^#^ *p* < 0.05, ^##^ *p* < 0.01 and ^###^ *p* < 0.001 compared with CP.

Previous studies have reported that increased accumulation of neutrophils, macrophages, and CD4^+^ T cells was observed in kidney tissues after cisplatin treatment [11,12]. As expected, cisplatin-injected mice displayed an increase in the numbers of cells stained with anti-Ly6B.2 (Figure 10A,B) or anti-galectin-3 (Figure 10C,D) antibody, compared with control mice. Interestingly, these changes were significantly alleviated by kahweol (Figure 10A–D). It has been well known that vascular adhesion molecules drive the recruitment of immune cells to inflamed tissues [2,3]. Thus, we next examined their renal expression levels in all experimental groups. We found that renal mRNA levels of E-selectin, vascular cell adhesion molecule-1 (VCAM-1), and ICAM-1 were markedly increased after cisplatin injection, which was significantly reduced by kahweol (Figure 10E). Western blot analysis also confirmed that increased ICAM-1 protein level after cisplatin injection was reduced by kahweol (Figure 10F,G).

## 4. Discussion

Cisplatin-induced nephrotoxicity, such as AKI, is a major hurdle in the clinical use of cisplatin. Because there has been no effective treatment for cisplatin-induced nephrotoxicity, the development of novel preventive strategies for preventing cisplatin-induced AKI is important and urgent. Recently, many researchers and pharmaceutical companies are paying much attention to the development of new drugs using substances derived from plants [29]. Kahweol is a natural diterpene extracted from coffee beans and has been shown to exhibit a therapeutic effect against several inflammatory diseases [15,16,17]. However, whether kahweol has a beneficial effect on cisplatin-induced AKI remains undetermined. In this study, we showed that administration of kahweol prevented the development of renal dysfunction and histopathological abnormalities in cisplatin-injected mice. Especially, tubular injuries were markedly attenuated by kahweol. Our findings suggest that kahweol has a renoprotective effect against cisplatin-induced functional and structural injury.

High levels of oxidative stress are one of the hallmarks of cisplatin-induced renal injury [1,2,3]. Cisplatin treatment induces excessive ROS generation in kidneys, leading to apoptotic death of tubular epithelial cells [6]. It has been shown that kahweol ameliorated liver injury induced by carbon tetrachloride in mice mainly through suppressing oxidative stress [23]. Kahweol also protected human dopaminergic neurons from oxidative stress-induced apoptosis [24]. In this study, we found that kahweol significantly suppressed cisplatin-induced oxidative stress and resultant tissue injury. TUNEL staining showed that a marked increase in tubular cell death after cisplatin treatment was significantly attenuated by kahweol. Caspase-3 activation was also inhibited by kahweol, indicating that kahweol attenuated cisplatin-induced apoptosis of tubular epithelial cells. Together, our findings suggest that kahweol inhibited oxidative stress and thereby inhibited tubular cell apoptosis in cisplatin-induced renal injury. In good agreement with these results, current evidence suggests that dietary antioxidants such as capsaicin, curcumin, quercetin, and resveratrol exert a protective effect against cisplatin-induced renal injury via inhibition of oxidative stress and apoptosis [30]. Importantly, these dietary antioxidants potentiate the anti-cancer action of cisplatin. Kahweol has been also shown to induce apoptosis in various types of cancer cells [31,32], indicating that the compound exerts different effects on normal cells and cancer cells.

In this study, we also found that kahweol suppressed NOX4 expression in the kidneys of cisplatin-injected mice. NOX4 is a major source of ROS in the kidney and plays a critical role in cisplatin-induced AKI [27,28]. Therefore, it seems that kahweol-induced downregulation of NOX4 mainly contributed to the suppression of oxidative stress in cisplatin-injected mice. Previous studies also have shown that cisplatin treatment reduced the expression and activity of antioxidant enzymes [5,33]. Consistent with these findings, we observed that mRNA and protein levels of MnSOD and catalase were largely decreased in the kidneys after cisplatin treatment. Importantly, this change was significantly inhibited by kahweol. Altogether, these findings suggest that kahweol inhibited cisplatin-induced oxidative stress by modulating pro-oxidant and antioxidant enzymes.

Necroptosis is a programmed form of necrosis and plays an essential role in AKI [34]. RIPK1, RPK3, and MLKL are key players in the process of necroptosis. Upon induction of necrosis, RIPK1 binds to RIPK3 and forms a multi-protein complex, leading to the phosphorylation and oligomerization of MLKL [34]. Oligomerized MLKL translocate to the plasma membrane and induces the membrane rupture and cell lysis. A previous study has demonstrated that cisplatin-induced renal injury was significantly attenuated in RIPK3 or MLKL knockout mice compared to wild-type mice [7]. Recent studies also reported that pharmacological inhibition of RIPK1 effectively attenuated necroptosis of tubular epithelial cells and renal injury in cisplatin-injected mice [35,36]. Consistently, we observed elevated renal expression of RIPK1, RIPK3, and p-MLKL after cisplatin injection. Interestingly, this change was significantly inhibited by kahweol, suggesting that the compound has an anti-necroptosis effect, in addition to the anti-apoptosis effect.

Inflammation also plays a crucial role in cisplatin-induced renal injury [1,2,3]. During cisplatin treatment, excessive amounts of cytokines are produced and secreted from infiltrated pro-inflammatory cells and tubular epithelial cells. It has been demonstrated that genetic or pharmacological suppression of TNF-α ameliorated cisplatin-induced AKI in mice [10]. In this study, we found that kahweol significantly reduces plasma levels of TNF-α and IL-6 in cisplatin-injected mice. Renal levels of cytokines were also decreased by kahweol. The compound also inhibited the NF-κB signaling pathway that plays an important role in the production of cytokines. Consistent with our findings, previous studies have shown that kahweol suppressed cytokine production and inflammatory responses in animal models of several inflammatory diseases [15,16,17]. It has been also known that immune cells, including neutrophils, macrophages, and CD4^+^ T cells, are infiltrated into injured kidneys, inducing additional damage [11,12]. In this study, we also observed that the number of neutrophils, macrophages, and CD4^+^ T cells were increased in the kidneys of cisplatin-injected mice compared to control mice. Kahweol significantly suppressed the excessive accumulation of these cells. We also found that increased expression of E-selectin, VCAM-1, and ICAM-1 after cisplatin injection was also inhibited by kahweol. These vascular adhesion molecules are primarily expressed in endothelial cells and promotes immune cell infiltration into the tissues [2,3]. In good agreement with our results, previous studies have reported that cisplatin treatment induced upregulation of vascular adhesion molecules in cisplatin-induced AKI [18,37]. Taken together, these results suggest that kahweol inhibited immune cell accumulation presumably through downregulation of vascular adhesion molecules. Further, when the plasma membrane ruptures during the necroptosis process, the contents of the cells leak, causing and exacerbating the inflammatory response [34]. Therefore, the suppressive effects of kahweol on cisplatin-induced necroptosis also may contribute, at least in part, to the inhibition of inflammatory responses.

## 5. Conclusions

In conclusion, these results suggest that kahweol protects against cisplatin-induced AKI through inhibiting oxidative stress, tubular cell death, and inflammation. Kahweol might be a potential preventive agent against cisplatin-induced AKI, enabling the high-dose use of cisplatin. Our data prompt clinical researchers to investigate the effect of kahweol against cisplatin-induced AKI in humans.

## Figures and Tables

**Table 1:** List of primers used in this study.

Gene	Primer Sequence (5′→3′)	Accession No.
NOX4 ^1^	Forward: GAACCCAAGTTCCAAGCTCATT Reverse: GGCACAAAGGTCCAGAAATCC	NM_015760
Catalase	Forward: CAAGTACAACGCTGAGAAGCCTAAG Reverse: CCCTTCGCAGCCATGTG	NM_009804
MnSOD ^2^	Forward: AACTCAGGTCGCTCTTCAGC Reverse: CTCCAGCAACTCTCCTTTGG	NM_013671
TNF-α ^3^	Forward: GACGTGGAACTGGCAGAAGAG Reverse: CCGCCTGGAGTTCTGGAA	NM_013693
IL-6 ^4^	Forward: CCAGAGATACAAAGAAATGATGG Reverse: ACTCCAGAAGACCAGAGGAAAT	NM_031168
E-selectin	Forward: AGCTACCCATGGAACACGAC Reverse: ACGCAAGTTCTCCAGCTGTT	NM_011345
VCAM-1 ^5^	Forward: CCCAGGTGGAGGTCTACTCA Reverse: CAGGATTTTGGGAGCTGGTA	NM_011693
ICAM-1 ^6^	Forward: TTCACACTGAATGCCAGCTC Reverse: GTCTGCTGAGACCCCTCTTG	NM_010493
GAPDH ^7^	Forward: ACTCCACTCACGGCAAATTC Reverse: TCTCCATGGTGGTGAAGACA	NM_001289726

^1^ Nicotinamide adenine dinucleotide phosphate oxidase 4. ^2^ Manganese superoxide dismutase. ^3^ Tumor necrosis factor-α. ^4^ Interleukin-6. ^5^ Vascular cell adhesion molecule-1. ^6^ Intercellular adhesion molecule-1. ^7^ Glyceraldehyde-3-phosphate dehydrogenase.

## References

[1] Holditch S.J., Brown C.N., Lombardi A.M., Nguyen K.N., Edelstein C.L. (2019). Recent Advances in Models, Mechanisms, Biomarkers, and Interventions in Cisplatin-Induced Acute Kidney Injury. Int. J. Mol. Sci..

[2] Pabla N., Dong Z. (2008). Cisplatin nephrotoxicity: Mechanisms and renoprotective strategies. Kidney Int..

[3] Sánchez-González P.D., López-Hernández F.J., López-Novoa J.M., Morales A.I. (2011). An integrative view of the pathophysiological events leading to cisplatin nephrotoxicity. Crit. Rev. Toxicol..

[4] Trujillo J., Molina-Jijón E., Medina-Campos O.N., Rodríguez-Muñoz R., Reyes J.L., Barrera D., Pedraza-Chaverri J. (2015). Superoxide anion production and expression of gp91(phox) and p47(phox) are increased in glomeruli and proximal tubules of cisplatin-treated rats. J. Biochem. Mol. Toxicol..

[5] Kim J.-Y., Park J.-H., Kim K., Jo J., Leem J., Park K.-K. (2018). Pharmacological inhibition of caspase-1 ameliorates cisplatin-induced nephrotoxicity through suppression of apoptosis, oxidative stress, and inflammation in mice. Mediat. Inflamm..

[6] Jiang M., Wei Q., Pabla N., Dong G., Wang C.Y., Wang T., Smith S.B., Dong Z. (2007). Effects of hydroxyl radical scavenging on cisplatin-induced p53 activation, tubular cell apoptosis and nephrotoxicity. Biochem. Pharmacol..

[7] Xu Y., Ma H., Shao J., Wu J., Zhou L., Zhang Z., Wang Y., Huang Z., Ren J., Liu S. (2015). A Role for Tubular Necroptosis in Cisplatin-Induced AKI. J. Am. Soc. Nephrol..

[8] Kim J.W., Jo J., Kim J.-Y., Choe M., Leem J., Park J.-H. (2019). Melatonin Attenuates Cisplatin-Induced Acute Kidney Injury through Dual Suppression of Apoptosis and Necroptosis. Biology.

[9] Tristão V.R., Pessoa E.A., Nakamichi R., Reis L.A., Batista M.C., Durão Junior Mde S., Monte J.C. (2016). Synergistic effect of apoptosis and necroptosis inhibitors in cisplatin-induced nephrotoxicity. Apoptosis.

[10] Ramesh G., Reeves W.B. (2002). TNF-alpha mediates chemokine and cytokine expression and renal injury in cisplatin nephrotoxicity. J. Clin. Invest..

[11] Miao N., Yin F., Xie H., Wang Y., Xu Y., Shen Y., Xu D., Yin J., Wang B., Zhou Z. (2019). The cleavage of gasdermin D by caspase-11 promotes tubular epithelial cell pyroptosis and urinary IL-18 excretion in acute kidney injury. Kidney Int..

[12] Miyagi M.Y.S., Latancia M.T., Testagrossa L.A., Andrade-Oliveira V., Pereira W.O., Hiyane M.I., Enjiu L.M., Pisciottano M., Seelaender M.C.L., Camara N.O.S. (2018). Physical exercise contributes to cisplatin-induced nephrotoxicity protection with decreased CD4+ T cells activation. Mol. Immunol..

[13] Poole R., Kennedy O.J., Roderick P., Fallowfield J.A., Hayes P.C., Parkes J. (2017). Coffee consumption and health: Umbrella review of meta-analyses of multiple health outcomes. BMJ.

[14] Ren Y., Wang C., Xu J., Wang S. (2019). Cafestol and Kahweol: A Review on Their Bioactivities and Pharmacological Properties. Int. J. Mol. Sci..

[15] Lee H.-F., Lin J.S., Chang C.-F. (2019). Acute Kahweol Treatment Attenuates Traumatic Brain Injury Neuroinflammation and Functional Deficits. Nutrients.

[16] Seo H.-Y., Kim M.-K., Lee S.-H., Hwang J.S., Park K.-G., Jang B.K. (2018). Kahweol Ameliorates the Liver Inflammation through the Inhibition of NF-κB and STAT3 Activation in Primary Kupffer Cells and Primary Hepatocytes. Nutrients.

[17] Kim J.Y., Kim D.H., Jeong H.G. (2006). Inhibitory effect of the coffee diterpene kahweol on carrageenan-induced inflammation in rats. Biofactors.

[18] Tanimura S., Tanabe K., Miyake H., Masuda K., Tsushida K., Morioka T., Sugiyama H., Sato Y., Wada J. (2019). Renal tubular injury exacerbated by vasohibin-1 deficiency in a murine cisplatin-induced acute kidney injury model. Am. J. Physiol.-Ren. Physiol..

[19] Dunn S.R., Qi Z., Bottinger E.P., Breyer M.D., Sharma K. (2004). Utility of endogenous creatinine clearance as a measure of renal function in mice. Kidney Int..

[20] Kim S.H., Jung G., Kim S., Koo J.W. (2018). Novel Peptide Vaccine GV1001 Rescues Hearing in Kanamycin/Furosemide-Treated Mice. Front. Cell. Neurosci..

[21] Kim J.-Y., Leem J., Jeon E.J. (2020). Protective Effects of Melatonin Against Aristolochic Acid-Induced Nephropathy in Mice. Biomolecules.

[22] Kim J.-Y., Leem J., Hong H.-L. (2020). Protective Effects of SPA0355, a Thiourea Analogue, Against Lipopolysaccharide-Induced Acute Kidney Injury in Mice. Antioxidants.

[23] Lee K.J., Choi J.H., Jeong H.G. (2007). Hepatoprotective and antioxidant effects of the coffee diterpenes kahweol and cafestol on carbon tetrachloride-induced liver damage in mice. Food Chem. Toxicol..

[24] Hwang Y.P., Jeong H.G. (2008). The coffee diterpene kahweol induces heme oxygenase-1 via the PI3K and p38/Nrf2 pathway to protect human dopaminergic neurons from 6-hydroxydopamine-derived oxidative stress. FEBS Lett..

[25] Kim J.-Y., Jo J., Kim K., An H.-J., Gwon M.-G., Gu H., Kim H.-J., Yang A.Y., Kim S.-W., Jeon E.J. (2019). Pharmacological Activation of Sirt1 Ameliorates Cisplatin-Induced Acute Kidney Injury by Suppressing Apoptosis, Oxidative Stress, and Inflammation in Mice. Antioxidants.

[26] Kim J.-Y., Lee S.-J., Maeng Y.-I., Leem J., Park K.-K. (2020). Protective Effects of Bee Venom against Endotoxemia-Related Acute Kidney Injury in Mice. Biology.

[27] Yang Q., Wu F.R., Wang J.N., Gao L., Jiang L., Li H.D., Ma Q., Liu X.Q., Wei B., Zhou L. (2018). Nox4 in renal diseases: An update. Free Radic. Biol. Med..

[28] Meng X.M., Ren G.L., Gao L., Yang Q., Li H.D., Wu W.F., Huang C., Zhang L., Lv X.W., Li J. (2018). NADPH oxidase 4 promotes cisplatin-induced acute kidney injury via ROS-mediated programmed cell death and inflammation. Lab. Invest..

[29] Lien E.J., Lien L.L., Wang R., Wang J. (2012). Phytochemical analysis of medicinal plants with kidney protective activities. Chin. J. Integr. Med..

[30] Gómez-Sierra T., Eugenio-Pérez D., Sánchez-Chinchillas A., Pedraza-Chaverri J. (2018). Role of food-derived antioxidants against cisplatin induced-nephrotoxicity. Food Chem. Toxicol..

[31] Oh S.H., Hwang Y.P., Choi J.H., Jin S.W., Lee G.H., Han E.H., Chung Y.H., Chung Y.C., Jeong H.G. (2018). Kahweol inhibits proliferation and induces apoptosis by suppressing fatty acid synthase in HER2-overexpressing cancer cells. Food Chem. Toxicol..

[32] Jeon Y.J., Bang W., Cho J.H., Lee R.H., Kim S.H., Kim M.S., Park S.M., Shin J.C., Chung H.J., Oh K.B. (2016). Kahweol induces apoptosis by suppressing BTF3 expression through the ERK signaling pathway in non-small cell lung cancer cells. Int. J. Oncol..

[33] Rjeibi I., Feriani A., Ben Saad A., Sdayria J., Saidi I., Ncib S., Souid S., Allagui M.S., Hfaiedh N. (2018). *Lycium europaeum* Extract: A New Potential Antioxidant Source against Cisplatin-Induced Liver and Kidney Injuries in Mice. Oxid. Med. Cell. Longev..

[34] Anders H.J. (2018). Necroptosis in Acute Kidney Injury. Nephron.

[35] Kuang Q., Xue N., Chen J., Shen Z., Cui X., Fang Y., Ding X. (2018). Necrostatin-1 Attenuates Cisplatin-Induced Nephrotoxicity Through Suppression of Apoptosis and Oxidative Stress and Retains Klotho Expression. Front. Pharmacol..

[36] Wang J.N., Liu M.M., Wang F., Wei B., Yang Q., Cai Y.T., Chen X., Liu X.Q., Jiang L., Li C. (2019). RIPK1 inhibitor Cpd-71 attenuates renal dysfunction in cisplatin-treated mice via attenuating necroptosis, inflammation and oxidative stress. Clin. Sci. (Lond.).

[37] Salem N., Helmi N., Assaf N. (2018). Renoprotective effect of platelet-rich plasma on cisplatin-induced nephrotoxicity in rats. Oxid. Med. Cell. Longev..

